# Piezo1 in neuropathic pain: a putative mechanotransduction hub and underlying mechanisms

**DOI:** 10.3389/fnmol.2026.1852195

**Published:** 2026-07-02

**Authors:** Kuangpin Liu, Chunyan Li, Wei Ma, Wenyu Zhao, Rongdi Sun, Yawen Zheng, Qinge Zhao, Jinwei Yang, Lechun Lyn

**Affiliations:** 1 Yunnan Province High-Altitude Hot Springs Medical and Health Care Key Laboratory, College of Rehabilitation, Kunming Medical University, Kunming, China; 2 Department of Neurology, The Second Affliated Hospital of Kunming Medical University, Kunming, China; 3 Institute of Neuroscience, Kunming Medical University, Kunming, China; 4 College of Basic Medical Sciences, Kunming Medical University, Kunming, China; 5 Second Department of General Surgery, First People's Hospital of Yunnan Province, Kunming, China

**Keywords:** calcium signaling, dorsal root ganglion, inflammatory mediator release, mechanosensitive membrane protein Piezo1, neuropathic pain

## Abstract

Neuropathic pain (NP) is a chronic pain syndrome caused by damage or dysfunction in the nervous system. Its pathogenesis involves enhanced neuronal excitability, inflammatory responses, and neuron–glial interactions. Mechanotransduction, a critical process converting physical forces into biochemical signals, significantly contributes to the initiation and maintenance of NP. Piezo1 is a novel mechanosensitive membrane protein widely expressed in the dorsal root ganglion (DRG). It mediates membrane tension perception and regulates intracellular calcium influx, influencing neuronal excitability and the release of inflammatory mediators. This review systematically summarizes the structural characteristics, activation mechanisms, downstream signaling pathways, and functional expression of Piezo1 in the DRG. The analysis was based on 112 high-quality articles retrieved from databases including CNKI, Wanfang, and PubMed published between 2022 and 2025. Special attention is given to the molecular mechanisms by which Piezo1 mediates neuron–glia interactions, and how mechanical sensitivity and neuroinflammation collectively drive NP development. Furthermore, this review discusses the therapeutic potential of Piezo1 antagonists and gene regulation strategies, highlighting its scientific and clinical significance as a promising therapeutic target. This review provides a theoretical basis for a deeper understanding of mechanotransduction in NP, promoting the development and application of mechanism-oriented interventions centered on Piezo1.

## Highlights

- Piezo1 redefines the mechanobiological paradigm of NP: By converting external and intrinsic mechanical stresses into Ca^2+^ signaling and multi-level downstream cascades, Piezo1 functions as a central molecular hub linking mechanical stimuli to neuronal excitability and inflammatory responses.
- Piezo1 drives a self-sustaining “mechanical-inflammatory-excitatory” feedback loop: Through coordinated regulation of neuron–glia interactions and signaling networks such as MAPK, NF-κB, and YAP/TAZ, Piezo1 amplifies the coupling between mechanosensitization and neuroinflammation, underpinning the persistence and progression of chronic NP.
- Piezo1 represents a mechanism-oriented therapeutic target for NP: Pharmacological and gene-regulatory strategies targeting Piezo1 offer a conceptual shift from symptomatic analgesia to precision, mechanism-based intervention for NP.

## 1 Introduction

Neuropathic Pain (NP) is a chronic pain state resulting from neurological injury or functional disorders. Its clinical manifestations include spontaneous pain, mechanical hyperalgesia, and allodynia. The pathogenesis of NP is highly complex, involving multiple pathological processes such as functional remodeling of ion channels, enhanced neuronal excitability, abnormal release of inflammatory factors, and interactions between neuroglial cells (Attal et al., 2023; McGinnis and Ji, 2023). Studies have shown that Peripheral Nerve Injury (PNI) significantly enhances the mechanical sensitivity of primary sensory neurons, particularly Dorsal Root Ganglion (DRG) neurons. This change is closely associated with increased intracellular calcium influx, neuronal depolarization, and decreased action potential thresholds (Sun et al., 2022). Furthermore, local inflammatory responses induced by PNI activate non-neuronal cells such as Satellite Glial Cells (SGCs), forming a highly pro-inflammatory microenvironment that further amplifies pain signaling (Li S. et al., 2022; Wong et al., 2024; Shin et al., 2023). With deeper research insights, increasing evidence suggests that mechanical forces not only induce PNI but also act as crucial driving signals sustaining chronic pain states (Ellis et al., 2022). Therefore, understanding how cells sense and transduce mechanical stimuli, particularly their conversion into persistent pain signals in the nervous system, is essential for elucidating NP mechanisms.

Mechanical signal transduction refers to the critical process through which cells translate external physical forces (e.g., compressive stress, tensile, or shear forces) into biochemical signals.

**Figure 1:**
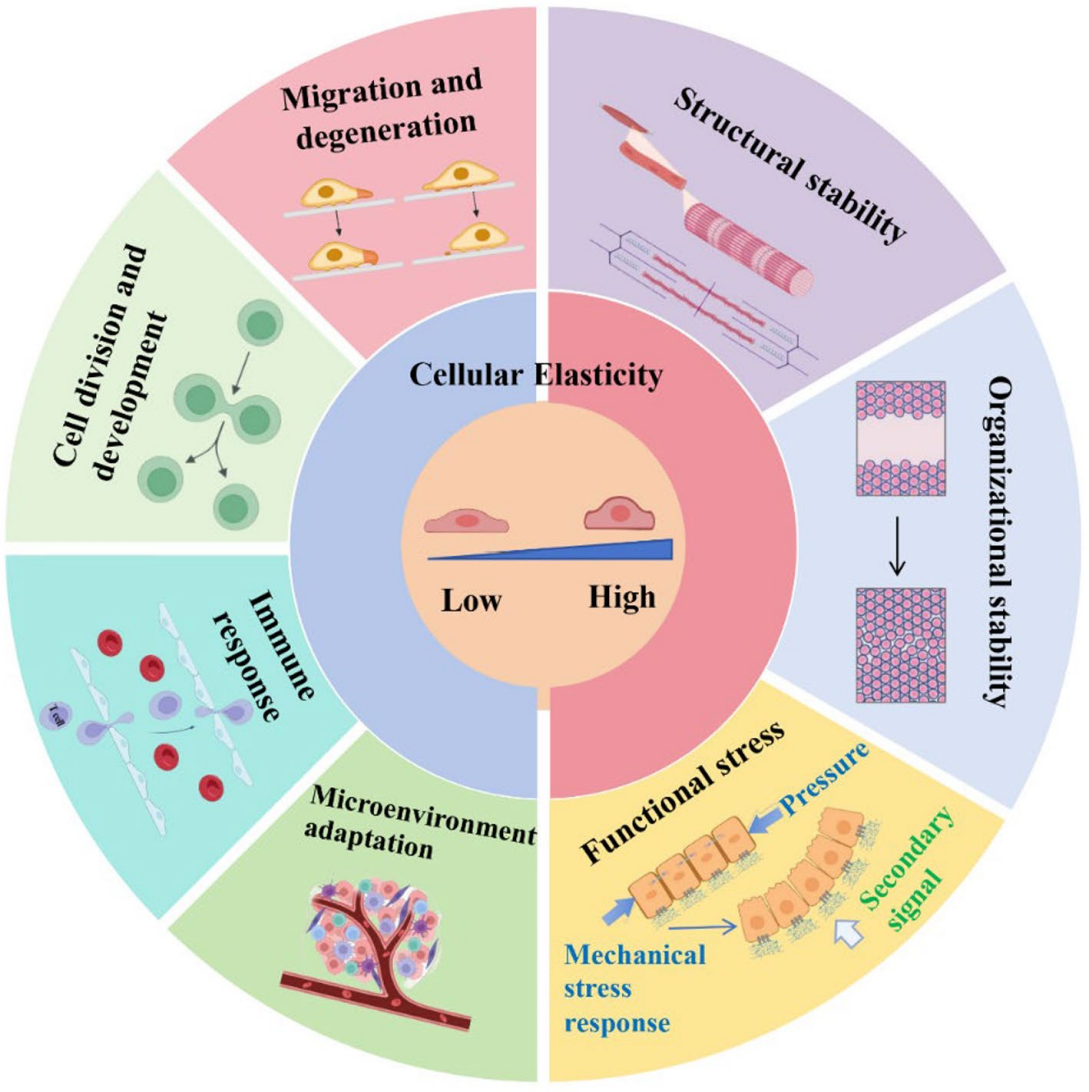
Schematic diagram illustrating the relationship between cellular elastic modulus and plasticity. The central panel shows the inverse relationship between cellular elasticity and plasticity. Surrounding panels illustrate seven functional outcomes of mechanical homeostasis: (1) Structural stability: cytoskeletal reinforcement in rigid cells; (2) Organizational stability: mechanically induced spatial ordering and dense packing of cells or molecular assemblies (circles represent self-organized cellular domains); (3) Functional stress response: transduction of external pressure into intracellular biochemical signals; (4) Microenvironment adaptation: mechanically driven vascular and stromal remodeling (tree-like structure indicates angiogenesis); (5) Immune response: mechanical regulation of immune cell activation; (6) Cell division and development: mechanosensitive control of proliferation and differentiation; (7) Migration and degeneration: mechanically directed cell motility and stress-induced degenerative changes.

Cells adapt to their environments by modulating their mechanical properties: cells with low elastic moduli generally exhibit greater plasticity and deformability, facilitating migration during development, tissue repair, and immune responses. For example, stem cells maintain flexibility to support differentiation, while immune cells utilize deformability to traverse blood vessels and perform defensive functions. In contrast, cells with high elastic moduli enhance cytoskeletal organization and matrix coupling to maintain structural stability, bear mechanical loads, or promote tissue repair, as observed in osteocytes, cardiomyocytes, and fibroblasts. Therefore, dynamic regulation of cellular stiffness reflects the functionality of mechanical signal transduction and represents an essential mechanism for maintaining mechanical homeostasis during development, injury repair, and disease progression (Di et al., 2023) (Figure 1). During this process, mechanosensitive ion channels serve as core “force sensors.” Their opening triggers transmembrane cation influx (e.g., calcium), rapidly activating downstream signaling pathways and regulating physiological processes such as cellular excitability, inflammatory responses, and gene expression (Miles et al., 2023; Kim and Hyun, 2023; Karska et al., 2023). Within the nervous system, these ion channels mediate mechanical sensations such as touch and hearing, and also critically participate in mechanical pain perception and signal amplification (Karska et al., 2023; Gudin et al., 2025). Multiple ion channel families, including the Piezo family (Piezo1 and Piezo2) and various transient receptor potential (TRP) channels (e.g., TRPV1, TRPA1, TRPM3, and TRPC1), have been identified as mechanosensitive or mechano-modulated contributors to somatosensation (Ji and Lee, 2021). While these channels collectively mediate mechanotransduction, their functional specialization and pathophysiological relevance to NP differ substantially. Piezo2 is well-established as the principal mechanotransducer for low-threshold, rapidly adapting touch sensation and proprioception (Woo et al., 2015). Its role is predominantly confined to physiological mechanosensation, and evidence directly linking Piezo2 to chronic NP pathogenesis remains limited. Similarly, TRPV1 and TRPA1 are primarily activated by chemical irritants and thermal stimuli, with mechanical modulation occurring mainly under acute pathological conditions rather than serving as a persistent driver of chronic pain states (Ji and Lee, 2021). By contrast, accumulating evidence suggests that Piezo1 occupies a distinct niche in NP pathophysiology. Following peripheral nerve injury (PNI), Piezo1 expression is markedly upregulated in DRG neurons and satellite glial cells (SGCs) (Shin et al., 2023), which directly implicated in NP initiation and maintenance. Unlike Piezo2, which mediates rapidly adapting responses to light touch, Piezo1 exhibits sustained activation under prolonged membrane tension—a biophysical property more consistent with the persistent mechanical hypersensitivity characteristic of chronic NP (Wan et al., 2024). Furthermore, Piezo1-mediated Ca^2+^ influx has been specifically coupled to downstream inflammatory signaling (e.g., NF-κB and MAPK pathways) and neuro-glial interactions (Malko et al., 2023), mechanisms that are central to NP chronicization but are less prominently associated with Piezo2 or TRP channels in this context. These differential expression patterns, biophysical properties, and signaling couplings raise the critical question of whether Piezo1 represents a distinct, non-redundant mechanosensory node in NP, or whether its effects merely reflect generalized mechanosensory perturbations common to multiple pathways (Pirri, 2025).

Structurally, Piezo1 is a large protein complex comprising 38 transmembrane helices, which undergoes conformational changes upon sensing membrane tension, rapidly activating the channel (Zhu J. et al., 2023). Functionally, Piezo1 is expressed in DRG neurons, vascular endothelial cells, and various glial cells, and it participates in regulating intracellular calcium homeostasis, inflammatory factor release, and neuro-glial signal transduction (Shin et al., 2023). Increasing evidence suggests that Piezo1 not only directly mediates mechanical pain perception but also contributes to the pathogenesis of NP by modulating inflammatory signaling pathways and other ion channels (Itson-Zoske et al., 2025; Wang H. et al., 2025). Therefore, Piezo1 is emerging as a promising therapeutic target in NP. Despite accumulating preclinical evidence implicating Piezo1 in neuropathic pain (NP) pathophysiology, its validation as a clinically actionable therapeutic target remains unfulfilled. First, all supportive data derive from rodent peripheral nerve injury models and *in vitro* systems. Few randomized controlled trial or prospective human study have established Piezo1 as a causal driver of chronic NP or confirmed that its modulation produces durable analgesia in patients (Xie et al., 2026). Second, the pharmacological toolkit is severely limited and confounded by poor selectivity—GsMTx4 broadly inhibits multiple mechanosensitive channels including TRPC1/6, while Dooku1 exhibits paradoxical agonist activity and cell-type-dependent potency, rendering interpretation of gain- and loss-of-function studies ambiguous (Thien et al., 2024). Third, genetic ablation strategies are compromised by robust compensatory mechanisms, as evidenced by upregulated Piezo2 and TRPV1 expression in sensory neurons following Piezo1 deletion, which obscures the channel’s non-redundant contribution to mechanical hypersensitivity (Ehlers et al., 2026). Fourth, Piezo1’s ubiquitous expression in vascular endothelium, erythrocytes, immune cells, and bone tissue raises substantial safety concerns regarding systemic inhibition, yet tissue-specific delivery systems remain undeveloped (Thien et al., 2024). Fifth, the structural complexity of this 38-transmembrane-helix mechanosensor and its intricate dependence on membrane lipid composition present formidable barriers to rational drug design (Lin Y. et al., 2025; Wang J. et al., 2025). Finally, the existing literature largely documents correlative associations between Piezo1 upregulation and pain behaviors without rigorously excluding the possibility that such changes represent downstream consequences rather than initiating pathogenic mechanisms (Wang J. et al., 2025).

This review assesses whether Piezo1 constitutes a distinct therapeutic candidate by systematically summarizing the structural and functional characteristics of Piezo1, elaborating on its molecular mechanisms in NP, and discussing its potential and prospects as a targeted therapeutic strategy, thereby laying the theoretical foundation for Piezo1 application in NP.

## 2 Materials and methods

### 2.1 Sources of information

A systematic literature search was performed by the first author from January 2022 to July 2025. This timeframe was selected on the rationale that the 2021 Nobel Prize in Physiology or Medicine, awarded to Ardem Patapoutian for the discovery of Piezo channels, catalyzed a substantial surge in mechanobiology research, rendering the 2022–2025 interval the most productive phase for mechanistic studies of Piezo1 in neuropathic pain.

Search Time Limit: The literature search included publications up to July 2025.
Search Databases: China National Knowledge Infrastructure (CNKI), Wanfang Data, Google Scholar, and PubMed.
Search Methods: Subject terms, keywords, and abstracts were searched.
Search Terms “Nerve injury,” “DRG,” “SGCs,” “DRG neurons,” “Neuroinflammation,” “NP,” “Mechanotransduction,” “Mechanosensitive ion channels,” “Piezo-type mechanosensitive ion channel component 1,” “mechanosensitive membrane protein Piezo1.”
Literature Retrieval Strategy: Search strategies for Chinese and English databases are presented in Table 1.
Literature Types: Reviews, experimental studies, and clinical studies were included.
Manual Search: References from recent and highly relevant cited articles were manually searched, read, and evaluated for inclusion criteria.
Literature Retrieval Volume: An initial search yielded 816 documents.

**Table 1:** Retrieval strategy.

Search term or phrase	Search type	Boolean logic
Nerve injury	MeSH+Title/Abstract	OR
DRG	MeSH+Title/Abstract	AND
SGCs	Title/Abstract	AND
NP	MeSH+Title/Abstract	AND
Mechanosensitive membrane protein Piezo1 OR Mechanosensitive ion channels OR Piezo-type mechanosensitive ion channel component 1	Title/Abstract	OR
Neuroinflammation	Title/Abstract	AND
Mechanotransduction	Title/Abstract	AND

### 2.2 Inclusion criteria

① Studies related to the role of mechanical signal transduction in NP; ② Studies investigating the mechanisms of NP mediated by the mechanosensitive ion channel Piezo1.

Exclusion criteria: Duplicate literature and studies not relevant to this topic.

### 2.3 Quality evaluation

Two independent researchers screened the titles, abstracts, introductions, and full texts of the retrieved literature according to the predefined inclusion and exclusion criteria. If discrepancies arose during the screening process, the two researchers discussed the issue and consulted a third reviewer to reach a final decision. According to the inclusion and exclusion criteria, a total of 112 studies were ultimately included in this review.

## 3 Results

### 3.1 Overview of Piezo channels

The Piezo channel family comprises two major members in vertebrates—Piezo1 and Piezo2—that function as non-selective mechanosensitive ion channels. Despite sharing a common architectural blueprint as large trimeric protein complexes with 38 transmembrane helices per subunit, these two paralogs exhibit markedly distinct biophysical properties, expression profiles, and physiological roles.

Piezo2 is well-established as the principal mechanotransducer mediating low-threshold, rapidly adapting touch sensation and proprioception (Ranade et al., 2014). Within the somatosensory system, Piezo2 is highly expressed in a subset of DRG neurons, particularly those innervating Merkel cells and Pacinian corpuscles, where it transduces innocuous mechanical stimuli into rapidly desensitizing electrical signals essential for light-touch discrimination and body-position awareness (Pirri, 2025). Its functional repertoire is predominantly confined to physiological mechanosensation and direct evidence linking Piezo2 to the initiation or maintenance of chronic neuropathic pain remains limited. Piezo1, by contrast, displays sustained activation under prolonged membrane tension and is widely distributed beyond the somatosensory system, including DRG neurons, satellite glial cells (SGCs), vascular endothelium, and various immune cells (Shin et al., 2023). Following peripheral nerve injury (PNI), Piezo1 expression is markedly upregulated in DRG neurons and SGCs (cell populations directly implicated in NP initiation and maintenance) (Shin et al., 2023). Unlike Piezo2, Piezo1 exhibits biophysical properties (slow inactivation and sustained Ca^2+^ influx) that align closely with the persistent mechanical hypersensitivity and ectopic firing observed in chronic NP. Furthermore, Piezo1-mediated Ca^2+^ signaling has been specifically coupled to downstream inflammatory cascades (e.g., NF-κB and MAPK pathways) and neuro-glial interactions that drive NP chronicization (Zhu J. et al., 2023; Malko et al., 2023), mechanisms that are less prominently associated with Piezo2 in pathological contexts. This clear functional difference between Piezo1 and Piezo2 explains why Piezo1 has become the main focus in NP research. Piezo2 mainly mediates acute touch and proprioception under normal conditions, whereas Piezo1 responds to sustained mechanical stress and connects this physical signal to inflammatory responses and neuronal hyperexcitability, making it more relevant to chronic pain.

#### 3.1.1 Structure and activation mechanism of Piezo1

Piezo1 is a large mechanosensitive ion channel protein and is among the largest ion channels identified to date. It consists of approximately 2,520 amino acids, with a molecular weight of about 250–300 kDa. In its functional state, Piezo1 assembles into a homotrimer in which three identical subunits are symmetrically arranged to form a three-bladed, propeller-shaped macromolecular complex (Saotome et al., 2018). It forms a macromolecular complex with a unique blade-like structure. Each subunit contains 38 transmembrane helices, which together form a central ion-conducting pore and peripheral mechanosensory modules. The pore region is composed of two terminal transmembrane helices, an extracellular cap structure, and an intracellular C-terminal domain. The peripheral mechanical sensing module includes beam-like structures, blade-like structures, and anchoring domains. This highly complex and dynamic spatial configuration enables Piezo1 to respond with high sensitivity to changes in membrane tension and various mechanical forces (Yang et al., 2022; Sugimoto et al., 2023).

**Figure 2:**
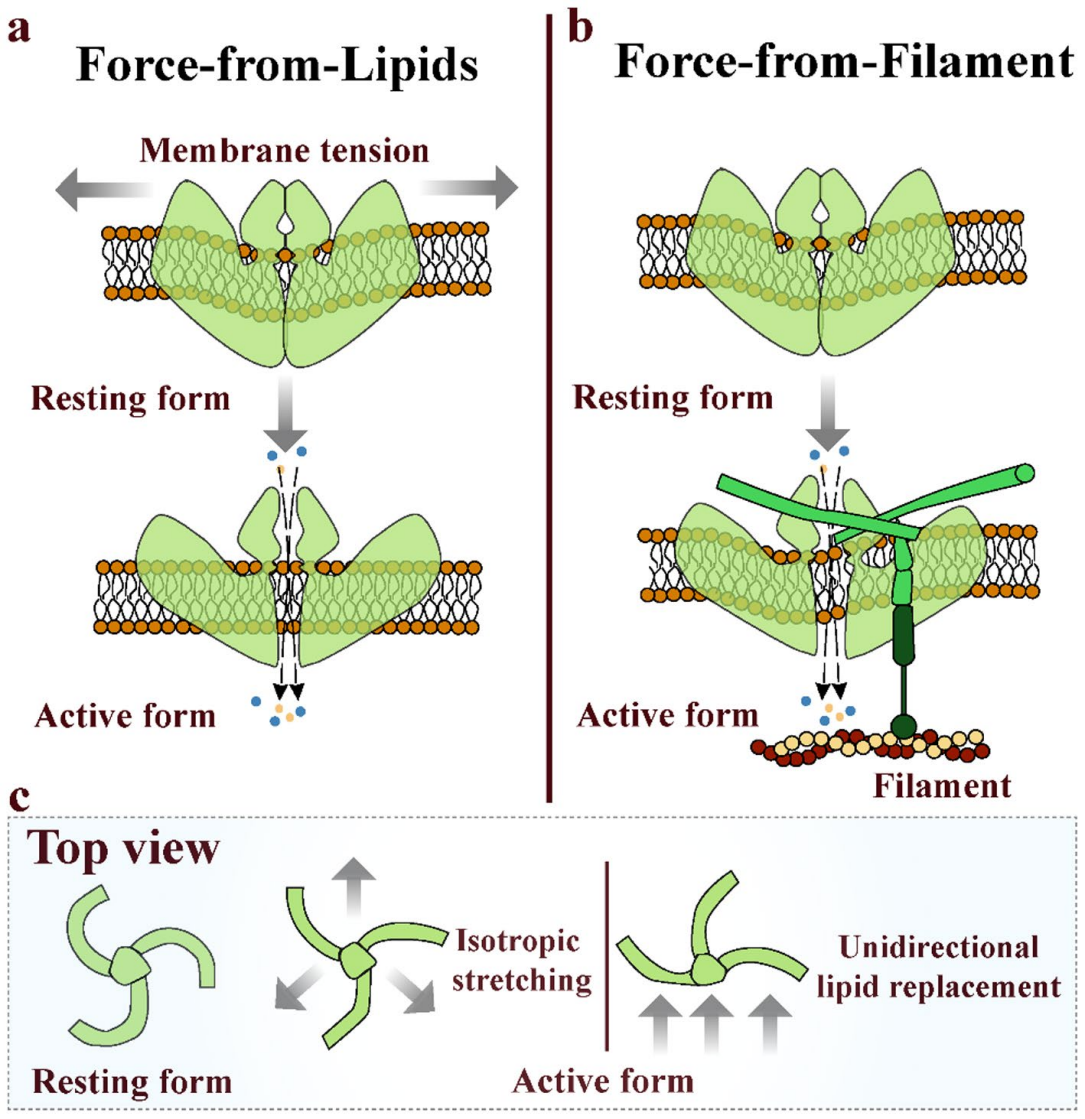
Activation mechanisms of Piezo1. **(a)** Force-from-Lipids model; **(b)** Force-from-Filament model; **(c)** Top view of Piezo1 (as a homotrimer).

Regarding its activation mechanism, two classical models have been proposed (Vasileva and Chubinskiy-Nadezhdin, 2023):

1. “Force-from Lipids” model: This model proposes that when Piezo1 is embedded in the lipid bilayer, it spontaneously forms an inwardly concave “dome-shaped” structure. As membrane tension increases, the curvature gradually flattens, leading to conformational changes and channel activation (Lacroix and Wijerathne, 2025) (Figure 2a). This concept is indirectly supported by animal studies conducted by Cheng et al. By knocking out the Piezo1 gene in vascular endothelial cells, they observed reduced cerebral blood flow, aggravated white matter lesions, and behavioral impairments, suggesting that Piezo1 regulates neurovascular function through sensing membrane tension (Ting et al., 2023).
2. “Force-from-Filament” model: This model emphasizes that Piezo1 activation depends on mechanical coupling with the cytoskeleton and the extracellular matrix (Wang H. J. et al., 2024) (Figure 2b,c). After Wang et al. disrupted filamentous actin (F-actin) structures or the β-catenin-E-cadherin complex in mice, they found that mechanically induced Piezo1 currents were markedly reduced, indicating that cytoskeletal tension plays a critical role in channel activation. In addition, the C-terminus of Piezo1 can interact with integrins. Integrin-mediated adhesion complexes exhibit spatial crosstalk with Piezo1, providing a molecular basis for extracellular mechanical forces to be transmitted to Piezo1 through anchoring proteins and thereby promoting its conformational activation (Cheng et al., 2023).

It is worth noting that the above two models are not mutually exclusive but function synergistically, thereby endowing Piezo1 with highly sensitive mechanosensing capabilities. While sensing membrane tension, Piezo1 also integrates cytoskeletal tension signals, thus mediating Ca^2+^ influx and regulating essential physiological functions such as cell volume, tension homeostasis, and endothelial permeability (Wang J. et al., 2022; Sforna et al., 2022). In the future, high-resolution cryo-electron microscopy, electrophysiological methods, and mechanical interventions at the cellular level are expected to further elucidate the fine structure and dynamic regulatory processes underlying Piezo1 activation.

#### 3.1.2 Downstream signal transduction of Piezo1

The C-terminal domain of Piezo1 significantly regulates Ca^2+^ influx and activates downstream signaling pathways. This Ca^2+^ signal not only influences intracellular homeostasis but also amplifies via cascade signaling mechanisms, activating multiple pathways such as Calcium/Calmodulin-dependent Protein Kinase II (CaMKII), Phosphatidylinositol 3-Kinase (PI3K)/Protein Kinase B (Akt), Mitogen-Activated Protein Kinase (MAPK), and Nuclear Factor kappa-B (NF-κB). These pathways critically regulate cellular proliferation, differentiation, apoptosis, inflammatory responses, and tissue remodeling (Lai et al., 2022).

##### 3.1.2.1 Ca^2+^-dependent signaling pathway

The increase in intracellular Ca^2+^ concentration represents one of the most direct cellular responses following Piezo1 activation. Ca^2+^ first binds to calmodulin (CaM) forming a Ca^2+^-CaM complex, thereby activating calcium/calmodulin-dependent protein kinases (CaMKs). CaMKs promote the activation of calcineurin phosphatase, which catalyzes the dephosphorylation of Nuclear Factor of Activated T-cells (NFAT), facilitating its nuclear translocation. Once inside the nucleus, NFAT acts as a transcription factor, regulating the expression of various downstream genes, particularly those involved in bone development and remodeling (Chen et al., 2023; Shindo et al., 2024).

Research by Ma et al. (2024) further demonstrated that Piezo1 activation induces Ca^2+^ influx, subsequently activating CaMKII, upregulating the expression of the osteogenic transcription factor Runx2, and promoting osteogenic differentiation and bone formation. This discovery provides a molecular basis for understanding how mechanical stress facilitates bone remodeling.

In addition, Piezo1-mediated Ca^2+^ signaling also activates the PI3K-Akt–mTOR pathway, which participates in regulating cell migration, survival, and metabolism (Chen and Zhang, 2024). Intracellular Ca^2+^ elevation activates PI3K, generating phosphatidylinositol (3,4,5)-trisphosphate (PIP_3_), which recruits and activates Akt at the plasma membrane. Akt, as a critical signaling node, controls multiple biological processes including cell cycle progression, apoptosis inhibition, glucose metabolism, and cellular motility. The downstream mammalian target of rapamycin (mTOR) further regulates protein synthesis, cell growth, and metabolic homeostasis (Shang et al., 2023; Xie D. G. et al., 2024; Li et al., 2024). In sciatic nerve injury models, studies have shown that Piezo1 regulates Schwann cell remyelination through the PI3K/Akt pathway, suggesting its potential role in peripheral nerve regeneration (Xu et al., 2025).

##### 3.1.2.2 MAPK signaling pathway

Activation of Piezo1 is often accompanied by phosphorylation of MAPK family members, including extracellular signal-regulated kinases (ERK1/2), p38 MAPK, and c-Jun N-terminal kinase (JNK) (Qiu et al., 2023; Xie B. et al., 2024). These signaling pathways play critical regulatory roles in cellular sensing and responses to mechanical stimuli, particularly in inflammatory diseases and within the tumor microenvironment (Qin et al., 2023). Piezo1-mediated activation of the ERK1/2 and p38 MAPK pathways is closely associated with the expression of pro-inflammatory factors such as IL-6 and TNF-*α*, thereby forming an important mechanism for the amplification of inflammatory signaling (Qin et al., 2023; Zhao C. et al., 2024).

In models of pulmonary fibrosis, Piezo1 induces Ca^2+^ influx after sensing mechanical stimulation, which subsequently activates hypoxia-inducible factor-1α (HIF-1α), upregulates TGF-β1 expression, and co-activates the MAPK- and SMAD-dependent pathways, thereby driving epithelial-mesenchymal transition and promoting fibrotic progression (Fang et al., 2023). Persistent and aberrant activation of this signaling pathway is considered a key pathological mechanism underlying chronic inflammatory diseases.

In addition, within the tumor microenvironment, the Piezo1-MAPK signaling axis can promote the migration, survival, and adaptation of tumor-associated cells to adverse microenvironmental conditions such as hypoxia and shear stress, and may mediate inflammation-related transcriptional reprogramming. Therefore, this signaling axis is regarded as an important link connecting mechanical forces, inflammatory responses, and tumor progression, and has considerable research and translational potential as a therapeutic target (Young and Reinhart-King, 2023).

##### 3.1.2.3 RhoA/ROCK pathway and cytoskeletal remodeling

Piezo1 can activate the small GTPase RhoA following mechanical stimulation, thereby initiating its downstream signaling pathway, Rho-associated coiled-coil-containing protein kinase (ROCK). The RhoA/ROCK pathway represents one of the core mechanisms regulating cytoskeletal remodeling. Activation of RhoA promotes the formation of actin stress fibers and enhances actomyosin contractility through ROCK-mediated myosin light-chain phosphorylation, thereby increasing cellular tension and adhesion. In addition, ROCK can regulate the activity of focal adhesion proteins, strengthening the interaction between cells and the extracellular matrix and further influencing cell polarity establishment and directional migration.

The Piezo1-RhoA/ROCK axis also plays a critical regulatory role in various physiological and pathological processes (Young and Reinhart-King, 2023). In epithelial cells, this pathway helps stabilize tight junctions and adherens junctions, thereby maintaining tissue barrier integrity (Berry et al., 2023). During tissue repair, it promotes the coordinated migration of epithelial cells or fibroblasts and accelerates wound healing (Berry et al., 2023). Within the tumor microenvironment, abnormal activation of this pathway can enhance the invasiveness and metastatic potential of cancer cells (Zhang et al., 2024). In summary, Piezo1 converts mechanical signals into dynamic cytoskeletal remodeling and migratory behavior through regulation of the RhoA/ROCK pathway, representing a key mechanism by which cells sense and respond to changes in the external mechanical environment.

##### 3.1.2.4 Hippo-YAP/TAZ pathway

The Hippo signaling pathway is a highly conserved intracellular regulatory network that primarily controls key biological processes such as cell proliferation, differentiation, and apoptosis through regulation of subcellular localization and activity of the transcriptional cofactors Yes-associated protein (YAP) and Transcriptional coactivator with PDZ-binding motif (TAZ). Under steady-state conditions, YAP/TAZ remains phosphorylated, leading to cytoplasmic retention or degradation by ubiquitination. Upon inactivation of the Hippo pathway, YAP/TAZ becomes dephosphorylated, translocates to the nucleus, and interacts with members of the TEA domain (TEAD) transcription factor family, inducing target gene expression (Yuan et al., 2025).

Piezo1 can indirectly influence the activity of Large tumor suppressor 1/2 (LATS1/2) by regulating cytoskeletal tension (e.g., stress fiber remodeling) and cell polarity, thereby modulating the phosphorylation levels of YAP/TAZ (Wang X. et al., 2024). Specifically, Piezo1 activation elevates intracellular Ca^2+^ concentration, activating the RhoA/ROCK signaling pathway, promoting F-actin polymerization and cytoskeletal remodeling, suppressing LATS1/2-mediated YAP/TAZ phosphorylation, facilitating nuclear translocation, and activating transcriptional programs. This mechanically induced activation of YAP/TAZ represents a critical mechanism by which cells perceive external mechanical cues and determine their fate (Berry et al., 2023).

In regulating stem cell fate, the Piezo1-YAP/TAZ pathway directs stem cell differentiation by precisely responding to mechanical signals such as cellular morphology changes and extracellular matrix stiffness (Koçak et al., 2024). Additionally, this pathway critically participates in tissue regeneration and organ development, influencing cell proliferation, tissue organization, and organ morphology (Lombardi et al., 2023). Notably, abnormal activation of this signaling axis in various solid tumors is closely associated with excessive tumor cell proliferation, epithelial-mesenchymal transition, increased resistance to apoptosis, and enhanced invasion and metastatic potential, thereby promoting tumor initiation, progression, and drug resistance (Peng H. et al., 2025).

##### 3.1.2.5 NO-sGC-cGMP pathway

The NO-sGC-cGMP pathway is a classical and vital signal transduction cascade involved in the vasodilatory response. In vascular endothelial cells, the mechanosensitive ion channel Piezo1 mediates increased intracellular Ca^2+^ concentration upon sensing mechanical stimuli such as shear stress. Elevated Ca^2+^ activates endothelial nitric oxide synthase (eNOS), catalyzing L-arginine conversion into nitric oxide (NO). As a critical gaseous signaling molecule, NO rapidly diffuses to adjacent vascular smooth muscle cells, binds to soluble guanylate cyclase (sGC), and converts it from an inactive conformation into an active form, catalyzing the generation of cyclic guanosine monophosphate (cGMP) from guanosine triphosphate (GTP).

As a second messenger, cGMP activates downstream effectors such as cGMP-dependent protein kinase (PKG), promoting intracellular Ca^2+^ sequestration and disassembly of actomyosin complexes, ultimately resulting in vascular smooth muscle relaxation, vasodilation, and improved local tissue perfusion. This pathway is central to physiological processes including vascular tone maintenance, blood pressure regulation, and adaptive responses to mechanical shear stress, and it is closely associated with the pathogenesis and progression of numerous cardiovascular diseases (Liu et al., 2023; Lim and Harraz OF, 2024).

##### 3.1.2.6 NF-κB inflammatory pathway

Under the synergistic effects of Ca^2+^ signaling and the MAPK pathway, Piezo1-mediated calcium signaling promotes the phosphorylation and degradation of the NF-κB inhibitory protein IκB, thereby activating the NF-κB signaling pathway and inducing the transcription of inflammation-related genes, including TNF-*α*, IL-1β, IL-6, COX-2, and iNOS (Liu et al., 2024b; Malko et al., 2023). In the nervous system, activation of the Piezo1-NF-κB signaling axis is closely associated with the pro-inflammatory phenotypic transformation of glial cells, which subsequently increases neuronal excitability and contributes to the initiation and maintenance of NP (Malko et al., 2023; Liu et al., 2024b; Zhang et al., 2023). In the intestine, this signaling axis participates in immune defense and inflammatory regulation by maintaining intestinal epithelial barrier integrity and mucosal immune homeostasis. Its abnormal activation is closely associated with the pathogenesis of inflammatory bowel disease (Li Z. et al., 2025). In addition, the Piezo1-NF-κB pathway also functions as an important downstream effector of various innate immune receptors, such as Toll-like receptors (TLRs) and NOD-like receptors (NLRs), serving as a key hub linking innate and adaptive immune responses (Sun et al., 2020).

### 3.2 The role and mechanism of Piezo1 in NP

#### 3.2.1 Expression and function of Piezo1 in DRG

**Figure 3:**
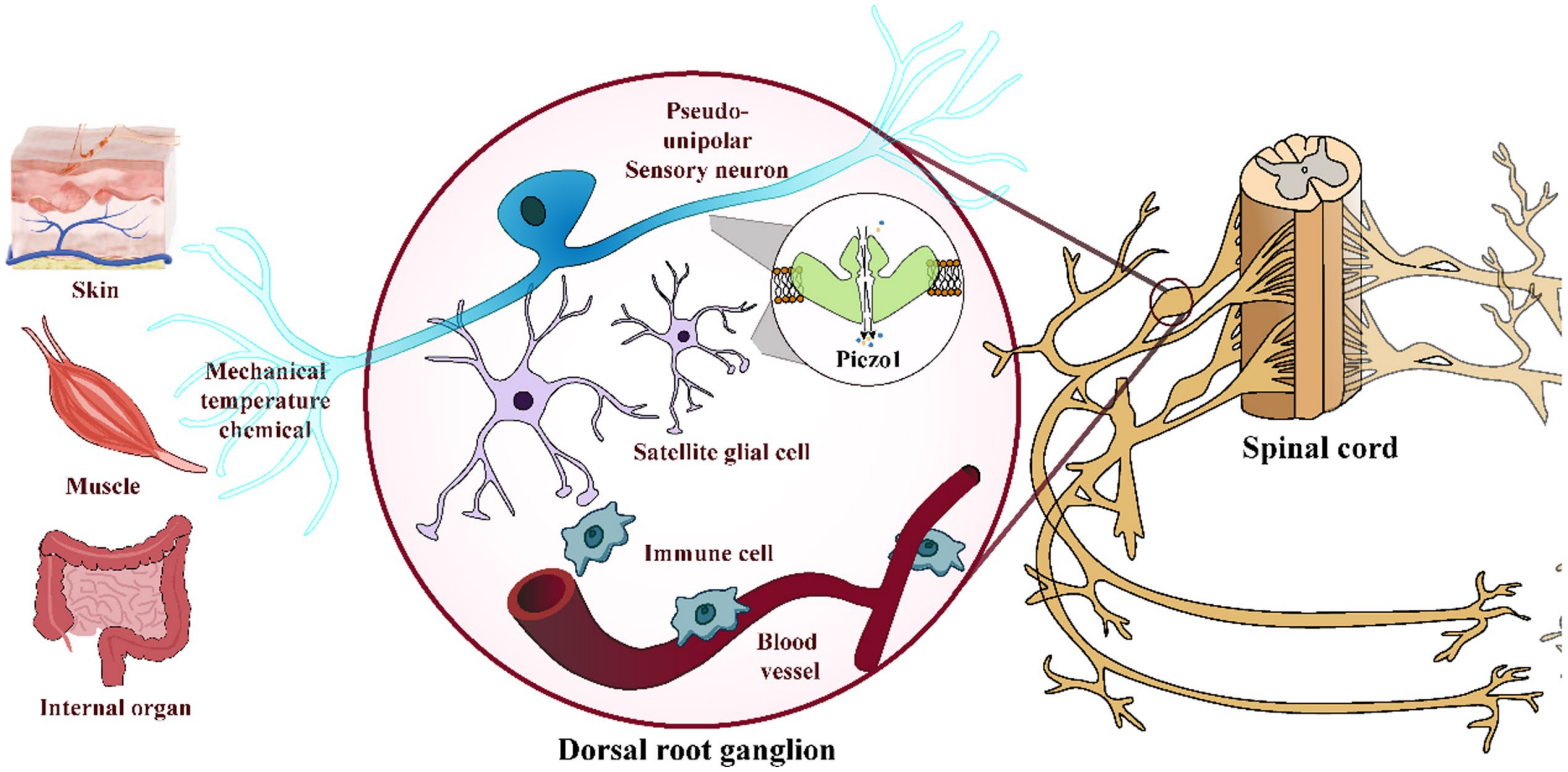
The dorsal root ganglion as a neuro-glial-immune sensory relay hub. Schematic of the DRG showing pseudounipolar sensory neurons surrounded by satellite glial cells and immune cells within a vascularized microenvironment, receiving convergent mechanical, thermal, and chemical inputs from skin, muscle, and visceral organs, and transmitting integrated signals to the spinal cord.

The DRG is a key sensory relay structure located on both sides of the spinal cord and belonging to the peripheral nervous system (PNS). Each DRG contains numerous pseudo-unipolar sensory neurons, which transmit mechanical, thermal, and chemical signals from the skin, muscles, and internal organs to the central nervous system (CNS). Because the DRG also contains non-neuronal components such as SGCs and immune cells, it forms a highly complex neuro-glial-immune microenvironment (Hao et al., 2023; Zhao Q. et al., 2024; Lian et al., 2024) (Figure 3).

Under pathological conditions such as nerve injury or inflammation, significant functional remodeling occurs in both neurons and glial cells within the DRG, which is considered a key event in the development of NP (Lian et al., 2024; Zhang et al., 2022; Tonello et al., 2023). NP often manifests as mechanical hyperalgesia and allodynia, and its underlying mechanisms involve increased neuronal excitability, ion channel remodeling, and enhanced release of inflammatory mediators (Denaro et al., 2024).

Recent studies have demonstrated that Piezo1 is expressed in DRG neurons of adult rats and mice and exhibits strong sensitivity to mechanical stimulation (Liu J. et al., 2024; Lee P. R. et al., 2024). Roh et al. confirmed through calcium imaging and molecular biological analyses that Piezo1 is predominantly distributed in small- and medium-sized DRG neurons, which are mainly nociceptive sensory neurons and frequently express channels such as Nav1.8 and TRPV1, both closely associated with pain transmission. In addition, Piezo1 expression has also been detected in SGCs and certain immune cells, suggesting that it may participate in neuron–glia interactions and inflammatory responses (Lee P. R. et al., 2024).

In NP models, Piezo1 activation may promote calcium wave propagation and inflammatory amplification in SGCs by regulating ATP release and activation of P2X/P2Y receptors, thereby further aggravating neuronal hyperexcitability. This “mechanical signal-calcium signal-inflammatory feedback loop” is considered one of the core mechanisms underlying mechanical hyperalgesia (Roh et al., 2020). Mechanistically, pathological membrane tension in injured DRG neurons opens Piezo1, triggering Ca^2+^ influx that lowers firing thresholds and promotes release of ATP and CGRP. These signals activate surrounding satellite glial cells (SGCs) via purinergic receptors, inducing secondary Ca^2+^ responses and release of pro-inflammatory cytokines (IL-1β, TNF-*α*). These mediators feed back onto DRG neurons to upregulate Piezo1 expression and enhance its membrane trafficking, further sensitizing the channel to mechanical stimuli. This creates a self-amplifying positive feedback loop in which neuronal and glial Piezo1 activities mutually reinforce one another, progressively locking the DRG into a hyperexcitable, pro-inflammatory state that underlies the persistence of mechanical allodynia. Inhibition of the Piezo1 channel has been shown to reduce mechanical hyperalgesia and spontaneous pain behaviors, providing experimental evidence supporting its therapeutic potential in NP (Ellis et al., 2022; Liu J. et al., 2024).

#### 3.2.2 Piezo1 mediates NP through neuron–glia interactions

**Figure 4:**
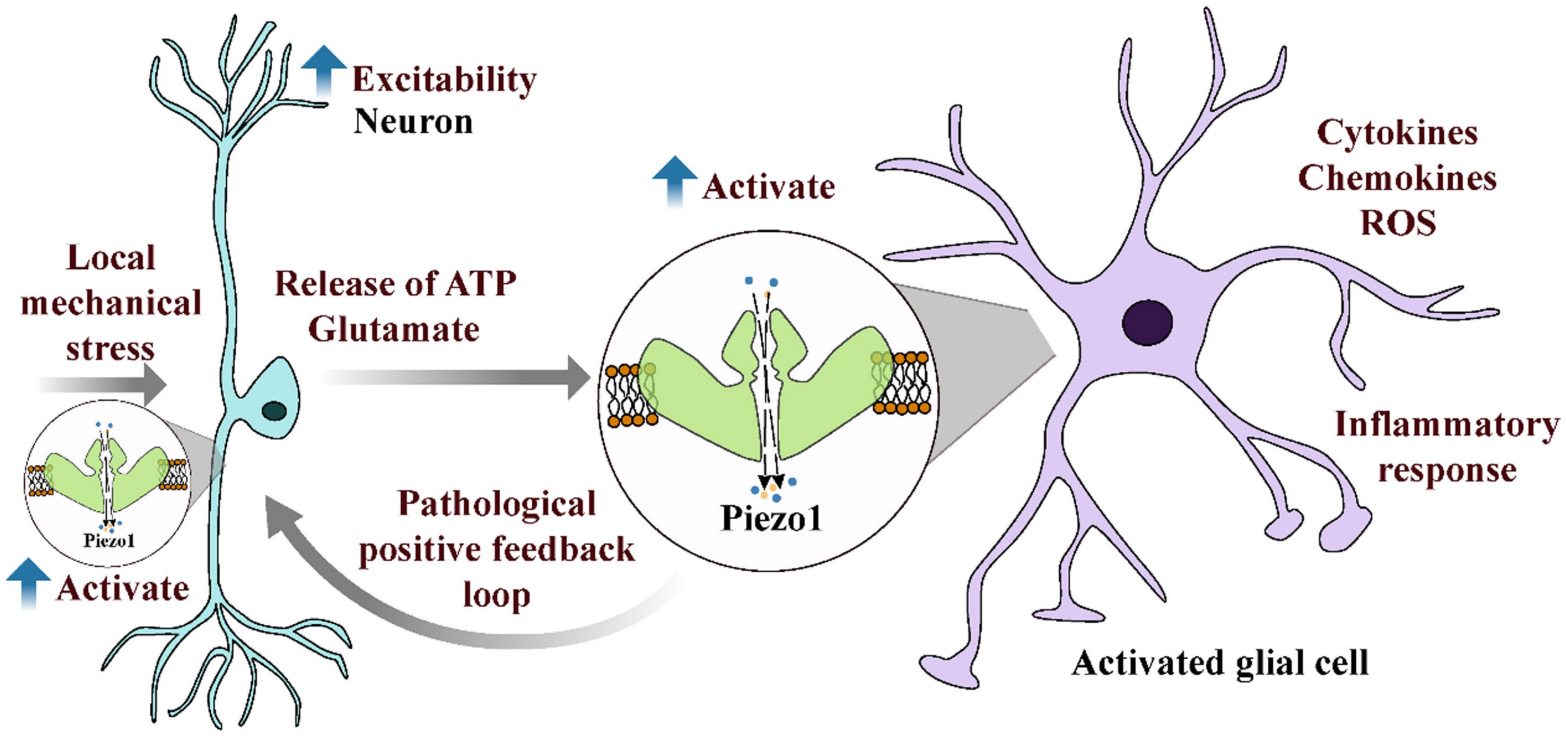
Pathological positive feedback loop between neurons and glial cells mediated by Piezo1. Piezo1-driven pathological positive feedback loop between neurons and glial cells in neuropathic pain. Local mechanical stress activates neuronal Piezo1, increasing excitability and promoting ATP/glutamate release. These signals activate adjacent glial Piezo1, triggering inflammatory responses (cytokines, chemokines, ROS). Released mediators further sensitize neurons, establishing a self-amplifying, pathological positive feedback loop between neurons and glial cells.

Piezo1 can convert local physical stress into initial intercellular signaling events (Ouyang et al., 2024). Under neuropathological conditions, increased neuronal excitability induces the release of signaling molecules such as ATP and glutamate, thereby activating Piezo1 channels in adjacent glial cells, triggering intracellular calcium oscillations and initiating inflammatory responses. Activated glial cells subsequently release cytokines, chemokines, and reactive oxygen species (ROS), which in turn act on neurons, creating a pathological positive feedback loop between neurons and glial cells, sustaining and amplifying abnormal signal transmission (Cho et al., 2023; Zhao et al., 2023) (Figure 4).

Under inflammatory conditions induced by lipopolysaccharide (LPS), Piezo1 expression in astrocytes is significantly upregulated. Activation of Piezo1 by Yoda1 induces intracellular Ca^2+^ oscillations and ATP-mediated signaling responses, while simultaneously suppressing the release of inflammatory factors IL-1β and TNF-*α* and inhibiting cell migration. This Piezo1-mediated “mechano–calcium–immune” signaling pathway regulates not only the functional state of glial cells themselves but also establishes bidirectional signaling interactions with neurons by altering glial secretion profiles: Ca^2+^-dependent release of ATP and glutamate modulates synaptic activity and neuronal excitability, whereas the reduced secretion of inflammatory factors helps alleviate neuronal toxicity, thereby maintaining synaptic stability and plasticity. Thus, Piezo1 serves as a critical channel through which astrocytes sense mechanical and inflammatory stress and regulate neuron–glia signaling interactions, highlighting its importance in understanding inflammation-mediated neural regulatory networks in the CNS (Yu et al., 2023).

In the PNS, when tissues experience mechanical stress (e.g., nerve compression or stretching) or are exposed to the Piezo1 agonist Yoda1, SGCs release paracrine signaling molecules such as ATP and glutamate via Piezo1-mediated Ca^2+^ influx. These signaling molecules activate channels, such as P2X receptors, on adjacent neurons, enhancing neuronal excitability and lowering action potential thresholds, thus intensifying mechanical hyperalgesia and establishing a “glial–neuronal sensitization linkage”. Through this Piezo1-dependent mechanotransduction mechanism, SGCs function not only as sensors of mechanical stress but also as active signal amplifiers that modulate neuronal plasticity and pain sensitivity, thereby highlighting significant therapeutic potential for NP (Shin et al., 2023).

#### 3.2.3 Piezo1-mediated mechanosensitivity and neuroinflammation synergistically drive NP

The initiation and maintenance of NP typically involve both heightened mechanical sensitivity and neuroinflammatory responses, forming a bidirectional and mutually reinforcing pathological mechanism mediated primarily through the mechanosensitive ion channel Piezo1 (Zheng et al., 2023) (Table 2).

**Table 2:** Piezo1-mediated mechanosensitivity and neuroinflammation synergistically drive NP.

Situation	The mechanism process	Ultimately manifests	Common scenarios related to NP
Inflammation activates mechanical ion channels → causes neuralgia	The release of inflammatory mediators (such as PGE_2_, IL-1β, TNF-α, etc.) → Alteration of the local environment (pH decrease, osmotic pressure change) → Direct or indirect activation of mechanical ion channels (Piezo1, TRPV4, ASICs, etc.) → Increased sensitivity of mechanical channels, and enhanced neural discharge under mechanical stimulation	Mechanical hyperalgesia is manifested as NP	NP, chronic inflammatory nerve injury (such as postherpetic neuralgia)
Mechanical ion channel activation → induction of inflammatory response → causing NP	Mechanical channels are activated (such as Piezo1, TRPV4, etc.) → calcium ion influx and membrane depolarization activate the MAPK/NF-κB signaling pathway → upregulation of inflammatory factors (IL-6, TNF-α, COX-2, etc.) causes local aseptic inflammation and intensifies pain	Persistent inflammation intensifies, pain sensitivity increases, and NP worsens	In the later stage of nerve injury, chronic mechanical load-related NP (such as chronic nerve compression syndrome)

On one hand, under conditions of nerve injury or chronic inflammation, immune cells and glial cells within the nervous system are activated, releasing large quantities of pro-inflammatory factors (Wang Q. et al., 2022). These factors can directly activate Piezo1, subsequently affecting the function of other pain-related ion channels, including Transient receptor potential vanilloid 1 (TRPV1), voltage-gated sodium channel Nav1.7, potassium channels, and Transient receptor potential ankyrin 1 (TRPA1). TRPV1 is a non-selective cation channel sensitive to heat, acidic environments, and capsaicin, primarily mediating pain signaling in sensory neurons. Studies have demonstrated that calcium influx induced by mechanical activation of Piezo1 not only directly initiates mechanical pain signaling but also modulates TRPV1 sensitivity through calcium-dependent signaling cascades (Lee P. R. et al., 2024). Roh et al. (2020) showed in mouse DRG neurons that Piezo1 activation promotes the phosphorylation of TRPV1, enhancing its responsiveness to mechanical and chemical stimuli and thus intensifying pain perception. This mechanism reveals cross-regulation between mechanical signaling and chemical pain pathways, clarifying the molecular basis underlying increased TRPV1 activity in mechanical hyperalgesia.

TRPA1, as a multifunctional channel responsive to both chemical and mechanical stimuli, is also involved in maintaining various pain states. Wan et al. indicated that mechanical activation of Piezo1 influences the conformation and activity of TRPA1 by modulating cell membrane tension. Furthermore, Piezo1-mediated calcium signaling activates downstream protein kinases, promoting TRPA1 phosphorylation and functional enhancement, thus forming a Piezo1-TRPA1 mechanical–chemical sensory amplification circuit and driving mechanical pain onset (Schumacher, 2024).

Voltage-gated sodium channel Nav1.7, widely distributed in the PNS, plays a crucial role in pain signal transduction. Changes in calcium influx and membrane potential mediated by Piezo1 can modulate Nav1.7 gating properties. Iseppon et al. confirmed that mechanical stimulation of Piezo1 accelerates Nav1.7 activation and delays its inactivation through membrane potential modulation, thereby increasing action potential frequency and enhancing pain signal transmission. This discovery provides novel insights into abnormal Nav1.7 activation in chronic NP (Wang Y. et al., 2024; Iseppon et al., 2024).

Potassium channels significantly regulate neuronal excitability, repolarization, and termination of action potentials. Although Piezo1 lacks direct binding to certain potassium channels (e.g., TREK1), it indirectly influences their opening probability through mechanically induced changes in membrane curvature and local ionic microenvironments (Lewis et al., 2024). Lai et al. (2022) reported that Piezo1 activation alters the membrane potential microenvironment, enhancing TREK1 activity, regulating neuronal excitability, and ultimately modulating pain signal intensity.

On the other hand, external mechanical stimuli such as traction, compression, or shear stress can directly activate Piezo1, triggering Ca^2+^ influx, initiating downstream inflammatory cascades, activating key signaling pathways such as MAPK and NF-κB, inducing synthesis and release of pro-inflammatory factors including TNF-*α* and IL-6, activating glial cells, enhancing neuronal excitability, and inducing aseptic neuroinflammation under non-infectious conditions (Cho et al., 2023). Such inflammatory responses not only heighten local neuronal sensitivity but also expand the affected area through mechanisms including extracellular ATP diffusion and gap junctions, promoting the progression from localized injury to network-wide pathological states (Xing et al., 2024). Concurrently, Piezo1 activation also synergistically modulates Nav1.7, TRPV1, and TRPA1 channel function, enhancing mechanical–chemical sensitization, improving neuronal integration of multisensory stimuli at the molecular level, and driving chronic NP progression (Tian et al., 2024).

In conclusion, these mechanisms do not operate independently; rather, they form a synergistic amplification and positive-feedback network orchestrated by Piezo1: inflammation enhances Piezo1 mechanosensitivity, while mechanical stress further intensifies inflammatory responses, ultimately forming an integrated chronic pain model characterized by “mechanical sensitization, inflammatory activation, neuronal excitation”. The Piezo1-mediated regulatory mechanism thus represents a unique pathway maintaining neuroinflammation, indicating that pain persistence is not solely due to initial injury but is maintained by a self-sustaining feedback loop between mechanical stress and inflammatory signaling.

### 3.3 Piezo1 as a potential therapeutic target for NP

#### 3.3.1 Application of Piezo1 antagonists

At present, no Piezo1-specific small-molecule antagonists have been approved for clinical use. However, several candidate compounds have demonstrated potential to modulate Piezo1 channel activity in both *in vitro* and *in vivo* studies, including GsMTx4, β-peptides, certain fatty acids, ruthenium red, gadolinium, Dooku1, as well as natural products such as berberine, tanshinone B, Acorus alkaloids, and ginsenosides (Ehlers et al., 2026). Among these, GsMTx4 is one of the most representative candidate antagonists. Derived from tarantula venom, it is a regulatory peptide that acts on mechanosensitive ion channels. It primarily inhibits Piezo1 activation indirectly by altering the mechanical tension transmission properties of the cell membrane, rather than directly blocking the ion-conducting pore. GsMTx4 belongs to the class of membrane-active peptides and can interact with the cell membrane in neutral or negatively charged lipid environments. By “softening” or stabilizing membrane structure, it reduces the transmission efficiency of mechanical forces and decreases the open probability of mechanically gated channels such as Piezo1 (Zhu Z. et al., 2023).

Animal studies have shown that local administration of GsMTx4 significantly alleviates mechanical hyperalgesia and spontaneous pain behaviors in models such as sciatic nerve ligation, diabetic neuropathy, and tumor-associated NP, without obvious motor side effects (Wang Y. et al., 2024; Ke et al., 2025; Bryniarska-Kubiak et al., 2023). However, GsMTx4 also exerts inhibitory effects on other mechanosensitive channels, including Piezo2 and TRPC6. Kinsella et al. reported that at a concentration of 5 μM, GsMTx4 suppressed Piezo1 currents by approximately 71–80%, Piezo2 by about 55%, TRPC5 by approximately 98%, and TRPC6 by around 70%, and also affected multiple voltage-gated sodium and potassium channels, indicating that it lacks high target specificity (Kinsella et al., 2024). Therefore, future studies should focus on optimizing its molecular structure to improve target selectivity. In addition, recent studies have attempted to integrate the GsMTx4 functional domain with nanocarriers or hydrogel delivery systems to enhance its targeting ability and *in vivo* stability, providing a promising direction for clinical translation (Pan et al., 2024).

#### 3.3.2 Gene regulation strategies targeting Piezo1

Compared with small-molecule antagonists, gene regulation strategies targeting Piezo1 offer greater specificity and longer-lasting effects, presenting significant therapeutic advantages in chronic diseases with persistent courses, such as NP (Tang et al., 2022). Current research primarily focuses on three main approaches: RNA interference (RNAi), microRNA (miRNA) modulation, and CRISPR/Cas9-mediated gene knockout (Lee M. et al., 2024).

Among these approaches, the adeno-associated virus serotype 9 (AAV9)—mediated shRNA delivery system is widely utilized to modulate Piezo1 expression in the spinal dorsal horn, DRG, and Schwann cells. For instance, researchers encapsulated shPiezo1 sequences into an AAV9 vector and administered it via intrathecal injection. In chronic sciatic nerve compression injury models, significant alleviation of mechanical hyperalgesia and inhibition of glial cell activation were observed, along with specific downregulation of Piezo1 in the DRG. These findings suggest that sensory neuron sensitivity remodeling might constitute a primary mechanism of action.

As important post-transcriptional regulators, microRNAs have emerged as critical modulators of Piezo1 expression. Previous studies demonstrated that miR-107 targets the 3′-UTR of Piezo1 mRNA, suppressing its translation. Under hyperglycemic conditions, miR-107 effectively mitigates Piezo1-mediated Ca^2+^ influx, neuronal hyperexcitability, and neuropathic alterations. Similarly, miR-223 negatively regulates Piezo1 indirectly in neuroinflammatory conditions, exerting analgesic and neuroprotective effects.

The CRISPR/Cas9 system is increasingly employed to achieve region-specific Piezo1 gene knockout, particularly in conditional knockout mouse models. Prior studies have confirmed that Piezo1 knockout in DRG neurons or Schwann cells significantly alleviates behavioral pain abnormalities caused by nerve injury, further validating its therapeutic feasibility (Mim et al., 2024; Hirai et al., 2014).

In the future, combining these precise gene regulation strategies with nano-delivery systems and tissue-specific promoters is expected to advance preclinical research and even personalized treatment applications (Mim et al., 2024). Furthermore, due to the complex multi-pathway and multi-cellular nature underlying NP pathogenesis, interventions targeting a single molecule typically fail to produce sustained analgesic effects.

### 3.4 Research Progress on Piezo1 and NP-related diseases

#### 3.4.1 Diabetic peripheral NP

Diabetic peripheral neuropathy is one of the most common chronic complications of diabetes, with 15–25% of diabetic patients developing NP (Jang and Oh, 2023). It frequently manifests as symmetrical and persistent burning pain, stabbing sensations, electric shock-like pain, tactile hypersensitivity, and hyperalgesia in the distal extremities, which typically worsen at night and severely impair patient sleep quality and daily life (Røikjer et al., 2024).

The pathological mechanisms underlying diabetic peripheral NP involve both peripheral and central sensitization. Peripheral sensitization primarily results from axonal degeneration of small nerve fibers (C and Aδ fibers), myelin sheath destruction, and upregulation of ion channels (such as NaV1.7 and TRPV1) in nerve endings under hyperglycemic conditions. Concurrently, the activation of non-neuronal cells, including glial cells, keratinocytes, macrophages, and other immune cells, induces inflammatory factor release, further exacerbating neuronal excitability and axonal injury (Røikjer et al., 2024; Gylfadottir et al., 2021; Hu et al., 2023). Regarding central sensitization, chronic abnormal pain signals maintain spinal dorsal horn neurons in a state of heightened responsiveness, accompanied by the activation of NMDA receptors and downregulation of GABAergic inhibitory pathways, thus causing exaggerated responses to normally innocuous stimuli or inducing spontaneous pain (Mishra et al., 2025). In addition, biomarkers associated with diabetic peripheral NP include TRPV1, GAP43, and Nav1.7, which exhibit markedly elevated expression under hyperglycemic conditions, contributing to oxidative stress and inflammatory responses (Røikjer et al., 2024). Recent studies have proposed that Piezo1 can be activated in response to hyperglycemia. Liu et al. demonstrated, using hyperglycemic mouse models and hyperglycemic HT22 cell models, that high glucose stress significantly upregulates Piezo1 expression. Activation of Piezo1 induces Ca^2+^ influx, subsequently activating CaMK2II, promoting oxidative stress and apoptosis, and ultimately causing neuronal synaptic damage and cognitive impairment. Furthermore, bezafibrate was found to inhibit Piezo1 expression through the PPARα/PANK1/miR-107 signaling pathway, thereby alleviating hyperglycemia-induced nerve damage and highlighting its therapeutic potential in diabetic neuropathy (Liu et al., 2024a).

#### 3.4.2 NP triggered by nerve injury

Traumatic spinal cord injury (SCI) is directly caused by mechanical forces, and the severity of injury depends on the type of mechanical insult, such as penetrating injury, traction, or compression trauma. Under mechanical stimulation, Piezo1 may participate in various pathological processes associated with SCI, including neuroinflammation, edema, neuronal death, and demyelination, ultimately contributing to NP (Zhang et al., 2025; Li Q. et al., 2025). Studies have demonstrated that Piezo1 expression is significantly upregulated following SCI, which may represent a cellular adaptive response to mechanical stress. As a mechanosensitive ion channel, Piezo1 regulates cellular functions by sensing external mechanical stimuli (Lv et al., 2024). The Piezo1 channel blocker GsMTx4 has shown pronounced neuroprotective and reparative effects, including promoting axonal regeneration, enhancing myelin formation, and reducing demyelination and neuronal damage in the CNS, thereby alleviating NP. Therefore, GsMTx4 has potential as a candidate therapeutic agent for SCI (Zhang et al., 2025).

Following PNI, the local neuromechanical environment is altered, activating a series of mechanical-electrochemical signaling pathways that influence nerve regeneration and functional recovery. Among these, the Piezo1-YAP/TAZ pathway is considered a key signaling mechanism involved in NP induction and myelin regeneration (Kong et al., 2022). Xu et al. reported that after sciatic nerve injury, interactions between growth cones and glial cells generate mechanical forces that may activate Piezo1 channels in Schwann cells. Combined treatment with massage therapy and GsMTx4 was found to modulate the mechanical microenvironment of the sciatic nerve. In addition, local mechanical stimulation can be detected by mechanosensitive ion channels and converted into electrical signals, thereby regulating signal transduction and tissue responses at the injury site (Xu et al., 2025; Zuela-Sopilniak and Lammerding, 2022; Wu et al., 2023; Shlapakova et al., 2024).

The NLRP3 inflammasome also plays a critical role in Piezo1-mediated chronic pain mechanisms. Li et al. found that sciatic nerve ligation activated the NLRP3 inflammasome, which subsequently promoted Piezo1-mediated inhibition of parvalbumin-expressing interneurons (PV-INs) in the anterior cingulate cortex (ACC). Upregulation of Piezo1 in PV-INs disrupts excitatory-inhibitory balance within the ACC neural network, contributing to the development and persistence of chronic pain. The ACC is a critical center for pain information processing. PNI can induce excessive neuronal excitation in this region and enhance synaptic plasticity of nociceptive neurons in the spinal dorsal horn. Administration of the NLRP3 inhibitor MCC950 effectively blocks caspase-1 and interleukin-1β expression induced by recombinant TNF-*α* (rrTNF) *in vitro*, significantly suppresses Piezo1 overexpression in the ACC following sciatic nerve injury, and markedly alleviates NP. These findings reveal the bridging role of the NLRP3-Piezo1 axis in chronic NP, providing new insights into the interaction between neuroinflammation and ion channel regulation and identifying potential therapeutic targets for pain management (Li Q. Y. et al., 2022).

#### 3.4.3 Chemotherapy-induced NP

Compared with the CNS, the PNS lacks structural protection such as the blood–brain barrier, making it more susceptible to direct toxic effects of anti-tumor drugs. These agents can trigger a series of inflammatory responses and facilitate the development and progression of chemotherapy-induced peripheral neuropathy (CIPN). Acute CIPN symptoms usually manifest within hours to days after drug infusion. Approximately 68% of patients still experience symptoms 1 month after chemotherapy ends, and about 30% continue to exhibit neurological dysfunction 5 months later (Was et al., 2022).

Studies have shown that certain chemotherapy drugs affect sensory neuron function by activating the mechanosensitive ion channel Piezo1, thereby inducing chronic NP (Was et al., 2022). Recent evidence indicates that epidermal keratinocytes play crucial roles not only in normal tactile perception but also in pathological mechanisms underlying paclitaxel-induced mechanical pain hypersensitivity. Experiments conducted on mouse chemotherapy models demonstrated that optogenetic or chemogenetic inhibition of keratinocyte activity significantly alleviated mechanical hypersensitivity from the second to the third week after paclitaxel exposure. Furthermore, paclitaxel treatment enhanced the mechanical sensitivity of mouse and human keratinocytes, simultaneously increasing endogenous Piezo1 currents. Additional research revealed that keratinocyte-specific knockout of Piezo1 markedly attenuated paclitaxel-induced mechanical hypersensitivity. These findings highlight the critical role of non-neuronal skin cells in chemotherapy-associated NP and provide an important theoretical foundation for developing novel analgesic strategies targeting keratinocytes and the Piezo1 channel (Mikesell et al., 2024).

#### 3.4.4 Low back pain accompanied by NP

Intervertebral disc degeneration (IVDD) is an important cause of low back pain (Jin et al., 2023). Mechanical stress has long been considered a key risk factor for IVDD onset, yet specific therapeutic targets addressing mechanical stress remain limited. Recent studies by Li F. et al. (2025). indicate that Piezo1 expression and activation are closely associated with IVDD progression. By constructing a mouse model with Piezo1-specific knockout, their research found significantly elevated Piezo1 expression in the nucleus pulposus tissues of degenerative intervertebral discs. Deletion of Piezo1 notably alleviated age- or mechanical instability-induced IVDD, reduced apoptosis, and diminished extracellular matrix degradation. Additionally, the Piezo1 antagonist GsMTx4 demonstrated considerable protective effects both *in vitro* and *in vivo*. Thus, targeting Piezo1 may delay or even reverse IVDD, offering a novel strategy for treating chronic low back pain (Li F. et al., 2025).

Piezo1 mediates IVDD through several specific mechanisms. First, Piezo1 channels sense mechanical stress, promoting iron ion influx and enhancing ferroptosis in nucleus pulposus cells, thereby contributing to IVDD (Xiang et al., 2024). Additionally, Piezo1 activation triggers Ca^2+^ influx, facilitates F-actin remodeling, and activates the YAP signaling pathway, accelerating pathological alterations in disc cells and IVDD progression (Peng F. et al., 2025). Under inflammatory conditions, Piezo1-mediated calcium influx activates CaMKII, promoting DRP1-mediated mitochondrial fission. This leads to mitochondrial dysfunction and energy metabolism disruption, ultimately exacerbating apoptosis and degeneration of cartilage endplate cells in intervertebral discs (Lin Z. et al., 2025; Ke et al., 2023). In addition, Piezo1 activates the NF-κB signaling pathway and induces expression of the extracellular matrix protein periostin. These changes collectively sustain inflammatory and senescence-related gene activation through self-amplifying cycles. Piezo1 also promotes cartilage endplate calcification, upregulates extracellular matrix-degrading enzymes (MMP-13 and ADAMTS-5), and triggers abnormal local immune microenvironments, collectively aggravating nucleus pulposus cell injury and structural degeneration of intervertebral discs (Wu et al., 2022).

## 4 Conclusion

NP has long posed challenges for basic research and clinical intervention due to its complex pathogenesis and highly heterogeneous clinical manifestations (Sachau and Baron, 2023). With the deepening understanding of pain mechanisms, increasing evidence suggests that mechanical stimulation is not only an external factor inducing injury but may also serve as a persistent driving force in the maintenance of chronic pain (Armstrong and Herr, 2025). As an important mechanosensitive receptor, the ion channel Piezo1 has been widely recognized as a “mechanotransduction hub” linking mechanical force input with biological signal responses because of its high sensitivity to membrane tension and elevated expression in DRG neurons and glial cells (Shin et al., 2023; Lüchtefeld et al., 2024).

However, current research on Piezo1 remains largely confined to basic studies, and several key issues still require urgent investigation. First, systematic comparisons of Piezo1 across different types of NP are lacking. Second, its activation mechanisms and interactions with other ion channels and inflammatory mediators remain incompletely understood. In addition, most existing studies rely on *in vitro* cellular models and animal experiments, with limited clinically relevant data available. Moreover, the absence of highly selective regulatory molecules targeting Piezo1 restricts the translation of current findings into clinical applications. Furthermore, the spatiotemporal dynamics of the Piezo1 signaling pathway and its stage-specific roles during pain progression have not yet been fully elucidated.

**Figure 5:**
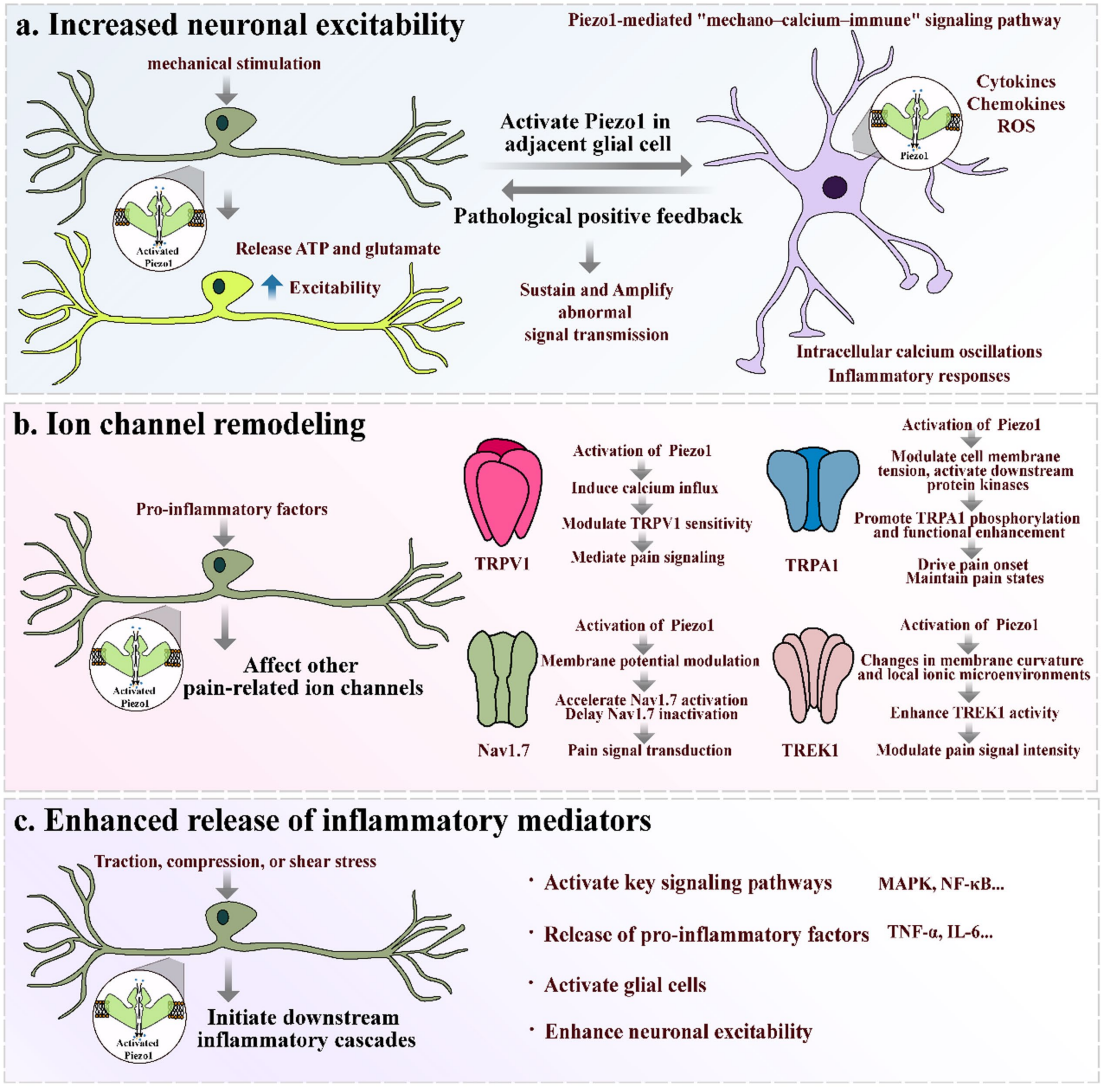
Proposed mechanisms of Piezo1 in neuropathic pain. Schematic overview of three convergent Piezo1-dependent mechanisms in NP: **(a)** increased neuronal excitability through neuron–glia positive feedback loops involving ATP/glutamate release and inflammatory amplification; **(b)** ion channel remodeling via modulation of TRPV1, TRPA1, Nav1.7, and TREK1 activity; and **(c)** enhanced release of inflammatory mediators through MAPK/NF-κB pathway activation and glial cell engagement.

Unlike traditional reviews that interpret NP from fragmented perspectives, such as inflammatory mediators, neuronal electrical activity, or single signaling axes, this article adopts mechanical signal transduction as the central framework and, for the first time, places the Piezo1 channel at the core of neuron–glia mechanical coupling. We propose that Piezo1 functions as a “mechanosensing-signal amplification-coupling” bridge, linking external mechanical forces with intracellular signaling cascades. This review not only systematically summarizes the mechanotransduction mechanisms of Piezo1 in different NP models but also elaborates on its interactions with classical inflammatory pathways, including MAPK and NF-κB, highlighting its role as a potential “signal integration node” in NP. These insights demonstrate strong theoretical expandability and promising prospects for targeted intervention strategies (Figure 5).

Overall, this review provides a novel mechanobiological perspective for understanding NP, while offering a theoretical foundation and technical direction for developing mechanism-oriented therapeutic strategies centered on Piezo1, with substantial research value and clinical translational potential. In the future, integrating knowledge of the spatially specific expression patterns of Piezo1 across different tissues and cell types with the development of precisely targeted molecular tools or drugs may promote a fundamental shift in NP treatment, from traditional symptomatic analgesia toward mechanism-based etiological intervention.

## Glossary

CaM: Calmodulin
CaMKII: Calcium/calmodulin-dependent protein kinase II
CIPN: Chemotherapy-induced Peripheral Neuropathy
CNS: Central nervous system
cGMP: cyclic Guanosine Monophosphate
DRG: Dorsal Root Ganglion
ERK1/2: Extracellular regulated protein kinases
F-actin: Filament actin
GAP43: Growth associated protein 43
IVDD: Intervertebral disc degeneration
LATS1/2: Large tumor suppressor 1/2
MAPK: Mitogen-activated protein kinase
NF-κB: Nuclear factor kappa-B
NFAT: Nuclear factor of activated T-cells
NO: Nitric Oxide
NP: Neuropathic Pain
PI3K: Phosphatidylinositol 3-kinase
Piezo1: Piezo-type mechanosensitive ion channel component 1
PNI: Peripheral Nerve Injury
PNS: Peripheral Nervous System
PV-INs: parvalbumin-expressing interneurons
ROCK: Rho-associated coiled-coil kinase
SCI: Spinal cord injury
SGCs: Satellite Glial Cells
sGC: soluble Guanylate Cyclase
TAZ: Transcriptional coactivator with PDZ-binding motif
TRPA1: Transient receptor potential ankyrin 1
TRPV1: Transient receptor potential vanilloid 1
YAP: Yes-associated protein
